# Role of salivary anti-SSA/B antibodies for diagnosing primary Sjögren’s syndrome

**DOI:** 10.4317/medoral.20199

**Published:** 2014-12-05

**Authors:** Pan Wei, Chunlei Li, Lu Qiang, Jing He, Zhanguo Li, Hong Hua

**Affiliations:** 1 Department of Oral Medicine Peking University School and Hospital of Stomatology, Beijing 100081, PR China; 2 Department of Rheumatology and Immunology, Peking University People’s Hospital, Beijing 100044, PR China

## Abstract

The diagnosis of primary Sjögren’s syndrome (pSS) is complex, and the saliva test is a potential method to improve the existing diagnostic criteria. Objective: To estimate the diagnostic accuracy of salivary anti-SSA/B antibodies in primary Sjögren’s syndrome (pSS), and to analyze their correlations with clinical and laboratory profiles. Study Design: This study enrolled 100 pSS patients and 140 non-pSS controls, including 40 rheumatoid arthritis (RA) patients, 40 systemic lupus erythematosus (SLE) patients, and 60 healthy controls. Unstimulated whole saliva and stimulated parotid saliva samples were collected from the subjects. Salivary anti-SSA/B antibodies were measured using an enzyme-linked immunosorbent assay (ELISA). Clinical and laboratory data were retrieved from the medical records. Results: In the pSS group, the sensitivity of anti-SSA and anti-SSB antibodies in whole saliva was 49% and 29%, respectively, and the specificity was 87.5% and 95%. The sensitivity of anti-SSA and anti-SSB antibodies in parotid saliva was 32% and 8%, respectively, and the specificity was 95.52% and 97.86%, respectively. In the pSS group, the diagnostic accuracy of anti-SSA/B antibodies in whole saliva was significantly higher than in parotid saliva (*p*<0.05), but was significantly lower than in serum (*p*<0.05). The salivary flow rate in the pSS group positive for whole salivary anti-SSA was significantly lower than in the negative group (*p*<0.05). The prevalence of rheumatoid factor and antinuclear factor were significantly higher in salivary SSB-positive pSS patients than in SSB-negative patients (*p*<0.05). Conclusions: Compared to parotid saliva, whole saliva is a more suitable diagnostic fluid. Using salivary anti-SSA/B antibodies as a single test item is insufficient given the relatively low sensitivity. Further studies should investigate the possibility of combining tests for different salivary autoantibodies as a method for diagnosing pSS.

** Key words:**Primary Sjögren’s syndrome, salivary diagnostics, anti-SSA autoantibodies, anti-SSB autoantibodies.

## Introduction

Sjögren’s syndrome (SS) is an autoimmune disease, characterized by lymphocytic infiltration and the destruction of exocrine glands, and resulting in a dry mouth (xerostomia) and dry eyes (xerophthalmia) (1). Exocrinopathy can occur alone as primary Sjögren’s syndrome (pSS) or in association with other autoimmune disorders (secondary SS), including rheumatoid arthritis (RA) and systemic lupus erythematosus (SLE) (2,3). Given the absence of gold standard diagnostic criteria, the early diagnosis and treatment of this disease is difficult (4,5). The diagnosis requires interdepartmental cooperation, including an assessment of salivary gland function, ophthalmological examination, serological tests, and a labial salivary gland biopsy. The serological test for SSA/B is usually indispensable, but is an invasive test.

Anti-SSA antibodies were initially found in patients with SS (6). They are among the antinuclear auto antibodies (ANA) detected most frequently, not only in SS, but also in other systemic autoimmune diseases, such as SLE, systemic sclerosis (SSc), myositis, and sometimes RA (7). Anti-SSA antibodies are detectable in 63% of pSS serum samples and in 46% of SLE samples (8), compared to only 3-15% of RA patients and 3-11% of SSA-positive SSc patients (9). There is a strong association between anti-SSA and anti-SSB antibodies. Anti-SSA can be found alone in many sera, while anti-SSB antibodies are usually accompanied by anti-SSA (10).

Recent studies have indicated that saliva obtained from SS patients can be tested for auto antibodies (11,12). However, the role that saliva auto antibodies play in the diagnosis of SS, the parallel relationship between saliva and serum auto antibodies, and their correlations with clinical manifestations remain unclear. This study investigated the salivary anti-SSA/B auto antibody levels in pSS patients and explored their value in the diagnosis of pSS.

## Material and Methods

The study participants were enrolled from the outpatient clinics of the Department of Oral Medicine, Peking University Stomatology Hospital, and the Department of Rheumatology & Immunology, Peking University People’s Hospital from 2007 to 2012. All of the pSS patients were diagnosed according to the revised international classification criteria (2002) for SS (13). In total, 100 pSS patients were recruited into the experimental group (95 females, 5 males; average age 54.23±13.44 years). The control group comprised 60 healthy individuals (43.2±11.00 years), 40 RA patients (53.3±11.28 years), and 40 SLE patients (40.9±11.32 years). The age and gender of the pSS and control groups were both matched. All of the SLE and RA patients were diagnosed according to the American College of Rheumatology (ACR) criteria for the classification of SLE (14) and RA (15), respectively. All of the study subjects gave informed consent before participating. Potential subjects were excluded if they smoked, had taken antibiotics, antifungals, or immunosuppressants within the previous 2 weeks, had a history of head/neck radiation or chemotherapy, hepatitis C virus (HCV) or human immunodeficiency virus (HIV) infection, lymphoma, amyloidosis, sarcoidosis, or graft-versus-host disease, or had taken anticholinergic drugs (e.g., atropine, hyoscyamine, propantheline bromide, belladonna).

Unstimulated whole saliva (WS) and parotid saliva (PS) samples were collected from all participants. The subjects were instructed to stop eating and drinking for 2 h before saliva collection. All collections were performed by the same dentist, in one place between 9:00 and 11:00 a.m., under similar light and temperature conditions. Before sampling, the participants were instructed to rinse orally for 1 min and to rest for 5 min. The WS was collected over at least 15 min and then centrifuged at 2600 × g for 15 min at 4°C. The supernatant was separated and stored at -80°C until subsequent use. Parotid saliva was collected using modified Carlson-Crittenden cups, which consist of two concentric circles of different diameters. A cup was placed over the buccal membrane during collection with the inner sucker aligned with the parotid duct orifice. Then, the suction cup was fixed to the buccal membrane by sucking the air out of the outer sucker. The PS was collected over a period of at least 15 min.

The salivary anti-SSA/B antibodies were tested using anti-SSA/B enzyme-linked immunosorbent assay (ELISA) immunoglobulin G (IgG) kits (EUROIMMUN, Lubeck Germany). The saliva samples were diluted 1:50 in phosphate-buffered saline (PBS), and 100 μL the diluent were used for the ELISA. The optical density (OD) was measured at 492 nm using an ELISA spectrophotometer (Bio-Rad Model 550; Microplate Reader, Hercules, CA, USA). Each sample was tested in triplicate. Positive values were defined as values greater than two standard deviations from the average OD measured in the control group.

Clinical and laboratory data were retrieved from the clinical records of all participants. The clinical information included age, gender, symptoms of dry mouth and dry eyes, results of Schirmer’s test, tear film break-up test, corneal staining test, parotid gland sialography, and labial salivary gland biopsy, and a history of other connective tissue diseases. The laboratory data retrieved included the serum anti-SSA/B, ANA, rheumatoid factor (RF), and IgG.

Data were analyzed using the SPSS 13.0 statistical software package. For dichotomous data, Pearson’s test and the McNemar test were used. For continuous data the t-test was used. P values less than 0.05 were considered statistically significant.

Results

1. General information of the participants.

Totally 100 pSS patients were recruited into experimental group, 95 female and 5 male, with an average age of 54.23±13.44 years old. Sixty healthy individuals, forty rheumatoid arthritis (RA) patients and forty systemic lupus erythematosus (SLE) patients were enrolled in control group, with average ages of 43.2±11.00, 53.3±11.28 and 40.9±11.32 years old, respectively. The age and gender were both matched between pSS group and control group.

2. Sensitivity and specificity of anti-SSA/B antibodies

in whole and parotid saliva.

The sensitivity of anti-SSA and anti-SSB antibodies in whole saliva was 49% and 29%, respectively, and the specificity was 87.5% and 95%, respectively. The sensitivity of anti-SSA and anti-SSB antibodies in parotid saliva was 32% and 8%, respectively, and the specificity was 95.52% and 97.86%, respectively ( Table 1). The prevalence of anti-SSA/B antibodies in whole saliva was significantly higher than in parotid saliva (*p*<0.05). The agreement between whole and parotid saliva anti-SSA was high, with a kappa value of 0.617 (*p*<0.05).

**Table 1:**
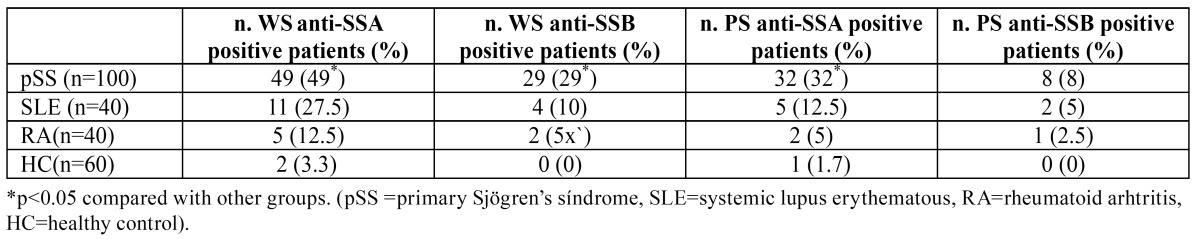
Prevalence of anti-SSA/B antibodies in whole saliva (WS) and parotid saliva (PS) in different groups.

3. Correlation between clinical features and anti-SSA/B antibodies.

There was a significant difference in the saliva flow rate between whole salivary anti-SSA-positive pSS patients and an-ti-SSA-negative pSS patients (*p*<0.05), whereas no significant differences were found in the other clinical parameters, including Schirmer’s test, the tear film break-up test, the corneal staining test, and parotid sialography examinations (all *p*>0.05) ( Table 2).

**Table 2:**
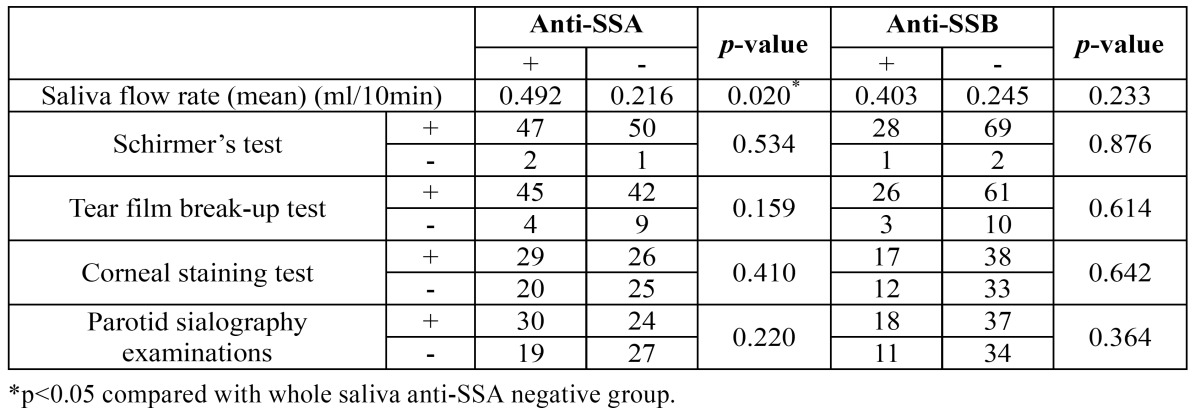
Comparison of clinical features in pSS patients with and without whole saliva anti-SSA/B antibodies.

4. Correlation between laboratory parameters and anti-SSA/B antibodies.

The positive rate of elevated IgG in whole salivary anti-SSA-positive pSS patients was significantly higher than in an-ti-SSA-negative pSS patients (*p*<0.05). The positive rates of RF and ANA in SSB-positive pSS patients were significantly higher than in the SSB-negative pSS patients (*p*<0.05) ( Table 3). In the pSS group, the diagnostic accuracy of anti-SSA/B antibodies in whole saliva was significantly lower than in serum (*p*<0.05). The agreement between serum and whole salivary anti-SSA was moderate, with a kappa value of 0.465 (*p*<0.05). The agreement between serum and whole salivary anti-SSB was high, with a kappa value of 0.720 (*p*<0.05).

**Table 3:**
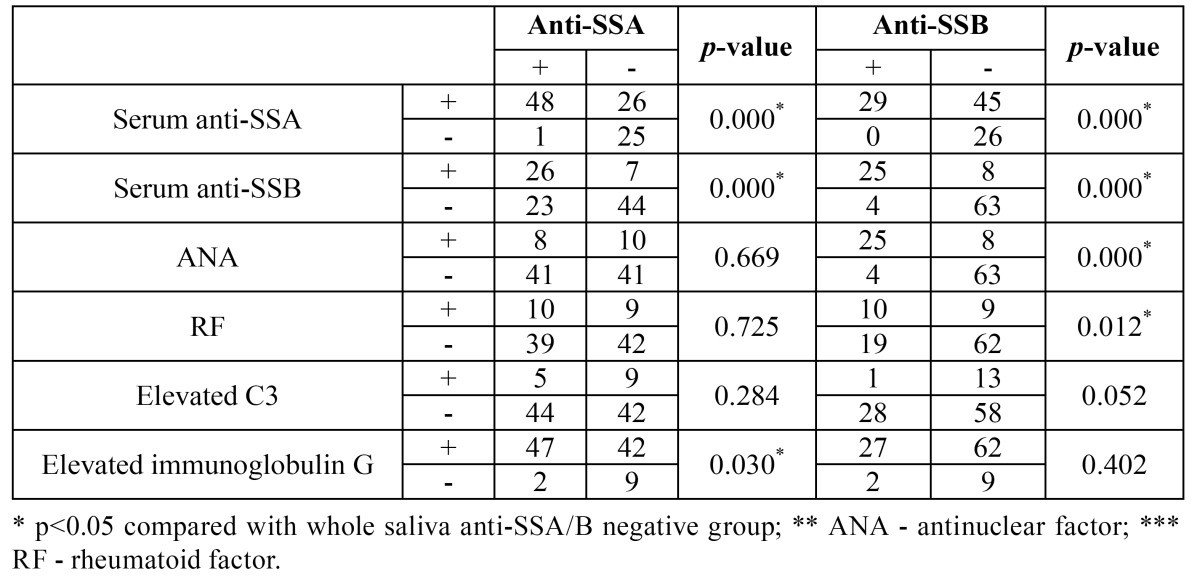
Comparison of laboratory parameters in pSS patients with and without whole saliva anti-SSA/B antibodies.

## Discussion

The expression of specific auto antibodies in the serum of patients with pSS is thought to be of great diagnostic value, with anti-SSA/B antibodies being the most represented (16). The prevalence of SSA and SSB ranged from 13-73% in the serum of patients with pSS (1). Compared to serum, the collection of saliva is noninvasive and convenient. Saliva is a potential tool for diagnosing SS. Because pSS mainly targets exocrine glands, salivary glands are directly and strongly influenced. As a result, the saliva test might have value in diagnosing the disease.

A decrease in saliva flow rate is a clinical characteristic of pSS, but the possible changes in the salivary constituents, such as SSA/B, are still unknown. Recently, researchers have detected several auto antibodies expressed in the saliva of patients with pSS, such as anti-alpha-fodrin, anti-M3R, and anti-SSA/B antibodies (17-19). Their roles in the diagnosis of SS have also been assessed. Sreebny (20) has studied the expression of anti-SSA/B antibodies in stimulated mixed saliva from 13 patients with SS, and reported that 46.15% were positive for salivary antibodies, with a specificity of 100%. However, the limitations of these studies, such as small sample size, and possible leakage of whole saliva, limit their value in determining the role of saliva testing in the diagnosis of SS.

Our study included 100 patients with pSS and 140 (60 healthy controls and 80 controls with other rheumatic diseases) controls. SSA/B antibodies were examined in both whole saliva and parotid saliva using ELISA. In the pSS group, the sensitivity of anti-SSA and anti-SSB antibodies in whole saliva was 49% and 29%, respectively, and the specificity was 87.5% and 95%, respectively. The sensitivity of anti-SSA and anti-SSB antibodies in parotid saliva was 32% and 8%, respectively, and the specificity was 95.52% and 97.86%, respectively. In the pSS group, the diagnostic accuracy of anti-SSA/B antibodies in whole saliva was significantly higher than in parotid saliva (p<0.05), but was significantly lower than in serum (p<0.05). The positive rate of salivary SSB was about 29%, with a poor correlation to serum SSB levels. Further studies are needed to interpret this result. Our results suggest that serum is significantly more sensitive than either whole or parotid saliva for detecting SSA/B. All patients who were positive for SSA/B in mixed saliva and parotid saliva also had serum that tested positive. Based on our results, serum leakage in mixed saliva seems to be the most likely explanation for the comparatively higher positivity in mixed saliva than parotid saliva.

Hammi *et al*. (21) studied anti-ENA antibody in parotid saliva and serum from 40 patients with SS, and reported that the positive rate of anti-ENA antibodies in serum was much higher than in parotid saliva. All of the patients with positive parotid salivary anti-ENA also had positive serum anti-ENA. Our findings are consistent with the study by Hammi *et al*. (21), which suggests a higher sensitivity and specificity of serum anti-ENA than saliva. Given the ease of collecting mixed saliva and the comparatively greater sensitivity and specificity, the use of whole salivary anti-ENA measurement in the diagnosis of SS is more practical than parotid saliva.

In comparing the clinical features of pSS patients with and without whole salivary anti-SSA/B antibodies, we found that the salivary flow rate in anti-SSA-positive pSS patients was significantly lower than in anti-SSA-negative pSS patients, whereas no significant differences were found in the Schirmer’s test, the tear film break-up test, the corneal staining test, or parotid sialography examinations between anti-SSA-positive and anti-SSA negative pSS patients. Atkinson *et al*. (22) reported that anti-SSA and anti-SSB antibodies can bond to normal parotid tissue by recognizing SSA/Ro and SSB/La antigens. Recently, Takada *et al*. (23) reported that the salivary flow rate in subjects with any of the antibodies associated with sicca (e.g., ACA, anti-SSA, anti-SSB) decreased with age. These results, combined with our findings imply that anti-SSA antibodies are possible to contribute to the salivary gland dysfunction. However, no differences were found in the clinical features of anti-SSB-positive and anti-SSB-negative pSS patients, which might be attributed to the very low positive rate of whole salivary anti-SSB antibodies in the participants.

In comparing the laboratory parameters in pSS patients with and without whole salivary anti-SSA/B antibodies, we found that three laboratory parameters were significantly (*p*<0.05) higher in anti-SSA-positive patients than in anti-SSA-negative patients; namely, serum anti-SSA, serum anti-SSB, and immunoglobulin G. In addition, four laboratory parameters were significantly higher in pSS patients with than in those without anti-SSB antibodies; namely, serum anti-SSA, serum anti-SSB, ANA, and RF. Our findings were partially consistent with Alexander *et al*. (10), who detected anti-SSA and anti-SSB antibodies more frequently in patients with RF, poly clonal hypergammaglobulinemia, and cryoglobulinemia. These results suggested that in the salivary anti-SSA/B positive pSS patients, hyper-immunoglobulin G, positive serum ANA and RF are more frequent. In addition, significant correlations were found between saliva and serum SSA/B, which suggests the suitability of whole salivary SSA in the diagnosis of SS.

The ELISA IgG kit was the only method used to analyze salivary anti-SSA and anti-SSB in our study. Other methods, such as immunodot and ELISA IgA kit, should be investigated, as they might have the potential to improve the sensitivity and specificity for salivary anti-SSA and anti-SSB antibodies.

In summary, the sensitivity and specificity of SSA/B in mixed saliva from patients with pSS was not as high as in serum. The positive rate of SSA/B in parotid saliva was significantly lower than in mixed saliva, whereas both had similar specificity, which is consistent with serum antibodies. Compared to parotid saliva, whole saliva is more suitable as a diagnostic fluid. Using salivary anti-SSA/B antibodies as a single test seems to be insufficient, given the relatively low sensitivity. Future studies should investigate the possibility of combining tests for different salivary auto antibodies as a method for diagnosing pSS.

## References

[1] Fox RI (2005). Sjögren's syndrome. Lancet.

[2] Tsuboi H, Asashima H, Takai C, Hagiwara S, Hagiya C, Yokosawa M (2014). Primary and secondary surveys on epidemiology of Sjögren's syndrome in Japan. Mod Rheumatol.

[3] Wakamatsu E, Nakamura Y, Matsumoto I, Goto D, Ito S, Tsutsumi A (2007). DNA microarray analysis of labial salivary glands of patients with Sjogren's syndrome. Ann Rheum Dis.

[4] Kruszka P, O'Brian RJ (2009). Diagnosis and management of Sjogren syndrome. Am Fam Physician.

[5] Kassan SS, Moutsopoulos HM (2004). Clinical manifestations and early diagnosis of Sjogren syndrome. Arch Intern Med.

[6] Anderson JR, Gray KG, Beck JS, Kinnear WF (1961). Precipitating autoantibodies in Sjogren's disease. Lancet.

[7] Menéndez A, Gómez J, Escanlar E, Caminal-Montero L, Mozo L (2013). Clinical associations of anti-SSA/Ro60 and anti-Ro52/TRIM21 antibodies: Diagnostic utility of their separate detection. Autoimmunity.

[8] Barakat S, Meyer O, Torterotot F, Youinou P, Briand JP, Kahn MF (1992). IgG antibodies from patients with primary Sjögren's syndrome and systemic lupus erythematosus recognize different epitopes in 60-kD SSA/Ro protein. Clin Exp Immunol.

[9] Franceschini F, Cavazzana I (2005). Anti-Ro/SSA and La/SSB antibodies. Autoimmunity.

[10] Alexander EL, Arnett FC, Provost TT, Stevens MB (1983). Sjogren's syndrome: association of anti-Ro (SS-A) antibodies with vasculitis, hematologic abnormalities, and serologic hyperreactivity. Ann Intern Med.

[11] Moody M, Zipp M, Alhashimi I (2001). Salivary anti-spectrin autoantibodies in Sjögren's syndrome. Oral Surg Oral Med Oral Pathol Oral Radiol Endod.

[12] Baldini C, Gallo A, Perez P, Mosca M, Alevizos I, Bombardieri S (2012). Saliva as an ideal milieu for emerging diagnostic approaches in primary Sjögren's syndrome. Clin Exp Rheumatol.

[13] Vitali C, Bombardieri S, Jonsson R, Moutsopoulos HM, Alexander EL, Carsons SE (2002). Classification criteria for Sjögren's syndrome: a revised version of the European criteria proposed by the American-European Consensus Group. Ann Rheum Dis.

[14] Hochberg MC (1997). Updating the American College of Rheumatology revised criteria for the classification of systemic lupus erythematosus. Arthritis Rheum.

[15] Arnett FC, Edworthy SM, Bloch DA, McShane DJ, Fries JF, Cooper NS (1988). The American Rheumatism Association 1987 revised criteria for the classifi cation of rheumatoid arthritis. Arthritis Rheum.

[16] Ben-Chetrit E, Fischel R, Rubinow A (1993). Anti-SSA/Ro and anti-SSB/La antibodies in serum and saliva of patients with Sjogren's syndrome. Clin Rheumatol.

[17] Busamia B, Gonzales-Moles MA, Mazzeo M, Linares J, Demarchi M, Gobbi C (2010). Assessing the determination of salivary electrolytes and anti-Ro and anti-La antibodies for the diagnosis of Sjögren's syndrome (SS). Med Oral Patol Oral Cir Bucal.

[18] Qin Q, Wang H, Wang HZ, Huang YL, Li H, Zhang WW (2014). Diagnostic accuracy of anti-alpha-fodrin antibodies for primary Sjögren's syndrome. Mod Rheumatol.

[19] He J, Qiang L, Ding Y, Wei P, Li YN, Hua H (2012). The role of muscarinic acetylcholine receptor type 3 polypeptide (M3RP205-220) antibody in the saliva of patients with primary Sjögren's syndrome. Clin Exp Rheumatol.

[20] Sreebny LM, Zhu WX (1996). The use of whole saliva in the differential diagnosis of Sjögren's syndrome. Adv Dent Res.

[21] Hammi AR, Al-Hashimi IH, Nunn ME, Zipp M (2005). Assessment of SS-A and SS-B in parotid saliva of patients with Sjogren's syndrome. J Oral Pathol Med.

[22] Atkinson JC, Royce LS, Wellner R, Pillemer SR, Bermudez D, Fox PC (1995). Anti-salivary antibodies in primary Sjögren's syndrome. J Oral Pathol Med.

[23] Takada K, Suzuki K, Okada M, Nakashima M, Ohsuzu F (2006). Salivary production rates fall with age in subjects having anti-centromere, anti-Ro, and/or anti-La antibodies. Scand J Rheumatol.

